# Relationship of JAK2 (V617F) Allelic Burden with Clinico-Haematological Manifestations of Philadelphia-Negative Myeloproliferative Neoplasms

**DOI:** 10.31557/APJCP.2020.21.9.2805

**Published:** 2020-09

**Authors:** Ka Shing Yow, Xin Liu, Chean Nee Chai, Moon Ley Tung, Benedict Yan, Dheepa Christopher, Kiat Hoe Ong, Melissa G Ooi

**Affiliations:** 1 *National University of Singapore, Singapore. *; 2 *Department of Haematology-Oncology, National University Hospital, Singapore. *; 3 *Department of Laboratory Medicine, Molecular Diagnosis Centre, National University Hospital, Singapore. *; 4 *Department of Haematology, Tan Tock Seng Hospital, Singapore. *

**Keywords:** JAK2, allelic burden, myeloproliferative disease

## Abstract

JAK2 (V617F) allelic burden is the main genetic driver behind and a potential differentiator between individual myeloproliferative neoplasm (MPN) subtypes. This study aimed to explore the relationship between JAK2 (V617F) allelic burden, MPN subtypes and their clinico-haematological manifestations in a Singapore-based cohort. Analysis was performed on a retrospectively collected dataset of 128 patients diagnosed with JAK2 (V617F) positive Philadelphia-negative MPNs between 2016 to 2017 in Singapore. Genomic analysis was conducted on blood samples via DNA extraction and Droplet Digital Polymerase Chain Reaction (ddPCR). The mean age was 62.4 (SD=14.1). 85 out of the 128 (66.4%) patients were male. There was a statistically significant difference in allelic burdens between the different MPN disease subtypes *χ*^2^(3) = 9.064, p=0.028, with essential thrombocytosis (ET) patients having the lowest mean JAK2 percentage allelic burden (26.5%). Patients with an allelic burden >50% had higher leukocyte counts (MWU 1016.5, p=0.001), haemoglobin levels (MWU 1287.0, p=0.045), lactate dehydrogenase levels (MWU 611.5, p=0.001), and lower platelet levels (MWU 1164.0, p=0.008). Subgroup analysis revealed none of these correlations was significant in the ET subgroup. The results are largely in concordance with previous research in Asian cohorts demonstrating the association between allelic burden and clinico-haematological manifestations of MPN. However, in the ET subgroup, the JAK2 (V617F) allelic burden do not correlate positively for haematological parameters which is only seen in Asian patients.

## Introduction

The discovery of a unique point mutation in exon 14 of JAK2 (V617F) in Philadelphia-negative myeloproliferative neoplasms (MPNs) was established by four independent groups in 2005 (Baxter et al., 2005; James et al., 2005; Kralovics et al., 2005; Levine et al., 2005). JAK2 (V617F) is a unique, recurrent acquired mutation involving a change of guanine to thymine at nucleotide 1849 in exon 14 which leads to a substitution of valine to phenylalanine at position 617 in the auto-inhibitory JH2 domain. This subtle mutant protein change results in a gain of function capable of activating the downstream signalling pathways, particularly the JAK-STAT pathway (Levine et.al., 2007), in the absence of cytokines and confers autonomous growth or hypersensitivity to cytokines. This mutation is found in around 95% of PV, 50-70% of ET and 40-50% of PMF patients. However, it is still unclear how the same mutation could be responsible for three different MPN phenotypes; Essential Thrombocytosis (ET), defined by an elevated platelet count but normal red cell mass; Polycythaemia rubra vera (PV), characterised by raised red cell mass and on occasion raised white cell counts; and Primary myelofibrosis (PMF), which manifests as peripheral cytopenia due to increased reticulin deposition in the bone marrow. 

Previous studies have explored the role of JAK2 (V617F) allelic burden as a potential differentiator between individual MPN subtypes (Park et al., 2013); highest levels were found in PV and the lowest in ET patients. Within individual MPN subtypes, JAK2 allelic burden was associated with a higher incidence of MPN symptoms, splenomegaly and stimulated haematopoiesis (Kerguelén et al., 2012; Vannucchi et al., 2011). JAK2 (V617F) allelic burden holds the potential for both diagnostic and prognostic purposes in this area. Although this has been established in Western cohort studies, there have been few studies to date exploring such correlation in an Asian population.

This study aimed to explore the relationship between JAK2 (V617F) allelic burden, MPN subtypes and their clinico-haematological manifestations in a Singapore-based cohort.

## Materials and Methods

*Methodology*

*Genomic analysis*

DNA extraction from blood was performed manually or using LabTurbo DNA Mini Kit. Droplet Digital Polymerase Chain Reaction (ddPCR) was used to detect the JAK2 mutation, which involved three main steps, namely droplet generation, PCR amplification and droplet reading. 

Droplet generation was achieved using a PCR mixture consisting of 2X ddPCR™ Supermix for Probes (no dUTP), FAM and HEX primer/probes and Alu I restriction enzyme, together with water and the sample. The mixture was placed into QX200^TM^ Droplet Generator, which uses Droplet Generation Oil and microfluidics to partition each sample into 20,000 nanoliter-sized droplets. 

The generated droplets were then transferred to a 96-well plate. PCR amplification was performed using a C1000 Touch^TM^ Thermal Cycler with conditions shown in Table 1. 

The plate containing the amplified products was then placed in a QX200^TM^ Droplet Reader, in which the samples were picked up and analysed using a two-colour detection system. Positive droplets that contained at least one copy for the target DNA molecule exhibited stronger fluorescence readings compared to negative droplets. The readings were analysed using QuantaSoft^TM ^Software which measured the number of positive and negative droplets for each fluorophore in each sample. 

*Statistical analysis*

Analysis was performed on a retrospectively collected dataset of 128 patients diagnosed with JAK2 (V617F) positive Philadelphia-negative MPNs between 2016 to 2017 in a combined cohort of patients from National University Hospital and Tan Tock Seng Hospital, Singapore. Informed consent was obtained for collection of the data in accordance to the National University Health System’s Domain Specific Review Board. All laboratory results and JAK2 (V617F) analysis were done at the point of diagnosis (+/- 2 weeks), before treatment was commenced. MPN definitions were based on the 2016 WHO diagnostic criteria for myeloid neoplasms. The clinical features, allelic burden and haematological parameters were tabulated at the point of diagnosis for each patient.

The information was analysed using IBM SPSS version 24 (IBM Corp., Armonk, NY, USA). Chi-squared test of independence was used to compare individual group characteristics and determine the association between allelic burden and clinical signs and symptoms. Comparison between MPN disease subtype haematological parameters was done using Mann-Whitney U test (2 groups) and Kruskal-Wallis test (>2 groups). Spearman’s correlation was used to analyse the correlation between allelic burden and hydroxyurea dose. The Kaplan-Meier method was employed to understand the survival distribution between high and low allelic burdens. Statistical significance was set at p < 0.05.

## Results

*Univariate analysis*

The mean age of the patient cohort was 62.4 (SD = 14.1). 85 out of 128 (66.4%) of the patients were male. By ethnicity, there were 90 (70.3%) Chinese, 18 (14.1%) Malay and 7 (5.5%) Indian patients. The percentage of MPN by diagnosis are as follows: 48.4% ET, 37.5% PV, 8.6% PMF, 5.5% MPN-U (myeloproliferative disease – unclassifiable).

*Comparing MPN subgroups*

Analysis of the cohort by MPN subtypes is summarised in Table 2.

Patients in the ET group were of a greater age than patients in the PV group *χ*^2^ (1) = 10.512, p = 0.001. PV patients were 1.94 times more likely to have splenomegaly compared to the ET group; *χ*^2^ (1) = 4.801, p = 0.028. However, there was no significant difference in gender *χ*^2^ (1) = 2.846, p = 0.092, MPN scores (p = 0.715), initiation of HU therapy (p = 0.348) or thrombotic events (p = 0.088) between the two groups.

PV patients had a significantly higher Hb count (MWU 361.5, p < 0.001) and lower platelet count (MWU 832.0, p < 0.001) compared to ET patients. There was no significant difference in the WBC count (MWU 1189.0, p = 0.101) or LDH count (MWU 685.0, p = 0.381) between these two groups.

*Relationship of allelic burden with clinico-haematological parameters*

Further analysis was carried out by splitting the cohort into two groups according to the JAK2 allelic burden; wild type and low allele burden defined by an allelic burden of >50%, and high allele burden by anything above (i.e. >50%).

**Figure 1:**
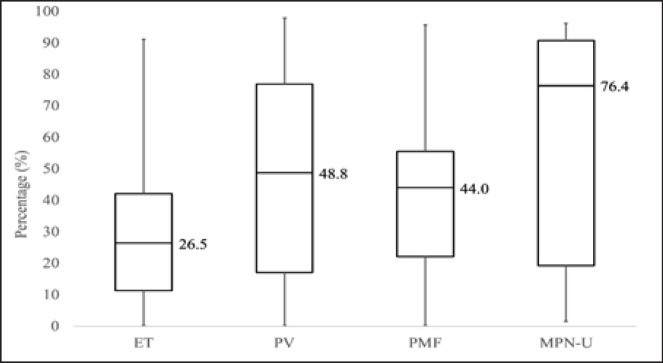
JAK2 Percentage Allelic Burden by MPN Diagnosis

There was a statistically significant difference in allelic burdens between the different MPN disease subtypes *χ*^2 ^(3) = 9.064, p = 0.028 (Figure 1). ET patients had the lowest mean JAK2 percentage allelic burden (26.5%), followed by PMF (44.0%), PV (48.8%), and MPN-U (76.4%) in ascending order.

Patients with an allelic burden >50% were more likely to present with splenomegaly *χ*^2^ (1) = 8.767, p = 0.003 (OR 3.7, 95% CI: 1.5 – 8.9). Allelic burden was not associated with a greater number of MPN10 symptoms *χ*^2^ (1) = 2.332, p = 0.127, thrombotic events *χ*^2^ (1) = 0.066, p = 0.798, or the initiation of hydroxyurea treatment *χ*^2^(1) = 0.337, p = 0.561.

Patients with an allelic burden >50% had higher leukocyte counts (MWU 1016.5, p = 0.001), Hb levels (MWU 1287.0, p = 0.045), LDH levels (MWU 611.5, p = 0.001), and lower platelet levels (MWU 1164.0, p = 0.008). Subgroup analysis for ET and PV patients was subsequently performed, revealing that the relationship between high JAK2 allelic burdens and the above-stated haematological parameters were not significant in the ET subgroup (Table 3).

*Thrombotic complications and cytoreductive therapy*

In terms of thrombotic complications, 43.0% (55/128) of the total cohort had previous arterial thrombosis and 3.9% (5/128) had previous venous thrombosis. Amongst the ET patients, 42.0% (26/62) have had a previous thrombotic event. This was 58.3% (28/48) in PV patients, 27.3% (3/11) in PMF patients and 42.9% (3/7) in the MPN-U group. No significant difference was seen between subgroups *χ*^2^ = 4.881 (p = 0.181).

There was a significant difference in prevalence of HU use amongst the different subgroups; ET 64.5% (40/62), PV 72.9% (35/48), PMF 9.1% (1/11), MPN-NOS 28.6% (2/7), *χ*^2^ = 18.730 (p < 0.001). This follows as HU is the mainstay therapy for the former two groups compared to the latter two. There was no difference in HU use between the ET and PV cohorts, as previously mentioned (p = 0.348). In the combined cohort, allelic burden was poorly correlated with hydroxyurea dose (Spearman’s value = 0.111).

*Co-existing mutations*

In this study, there was one patient with co-existing MPL positive and two patients with co-existing CALR positive (total 2.34% – 3/128). The allelic burden of the MPL positive patient was 0.27%, and that of the CALR positive patients were 39.6% and 76.6% respectively.

*Follow-up*

A log rank test using the Kaplan-Meier method done from the point of MPN diagnosis to July 2020 showed there was no significant difference between the survival distribution of the group with low allelic burden compared to that of high allelic burden, *χ*^2^ (1) = 0.57 (p = 0.812).

**Table 1:** Parameters Used for the C1000 Touch^TM^ Thermal Cycler

Cycling step	Temperature (^o^C)	Time	Ramp rate (^o^C/second)	No. of cycles
Enzyme activation	95	10 min	~2	1
Denaturation	94	30 sec		40
Annealing/extension	55	1 min		
Enzyme deactivation	98	10 min		1
Hold	4	Infinite	~1	1

**Table 2:** Cohort Statistics by MPN Subtypes

Characteristics	ET	PV	MF	MPN-U	Total
Demographics					
No. of patients	62 (48.4%)	48 (37.5%)	11 (8.6%)	7 (5.5%)	128 (100.0%)
Age in years (SD)	63.7 (15.9)	57.8 (11.5)	68.9 (11.1)	71.7 (6.8)	62.4 (14.1)
Male	37 (59.7%)	36 (75.0%)	8 (72.7%)	4 (57.1%)	85 (66.4%)
Chinese	45 (72.6%)	33 (68.8%)	7 (63.6%)	5 (71.4%)	90 (70.3%)
Malay	8 (12.9%)	7 (14.6%)	2 (18.2%)	1 (14.3%)	18 (14.1%)
Indian	4 (6.5%)	3 (6.3%)	0 (0.0%)	0 (0.0%)	7 (5.5%)
Biochemical & Lab Tests					
WBC count median (IQR)	11.3 (9.1 - 14.1)	13.1 (9.8 - 17.2)	10.6 (7.7 - 23.0)	13.7 (10.6 - 20.2)	11.7 (9.1 - 15.9)
Hb median (IQR)	13.6 (12.7 - 14.9)	17.4 (16.1 - 18.9)	9.4 (8.9 - 11.0)	12.1 (9.9 - 13.5)	14.4 (12.6 - 16.8)
Platelet count median (IQR)	754 (636 - 964)	538 (359 - 723)	311 (129 - 435)	566 (86 - 776)	669 (451 - 884)
LDH median (IQR)	635 (440 - 803)	688 (491 - 879)	1323 (968 - 1669)	1076 (809 - 1307)	702 (496 - 953)
MPL	0 (0.0%)	0 (0.0%)	1 (9.1%)	0 (0.0%)	1 (0.8%)
CALR	1 (1.6%)	1 (2.1%)	0 (0.0%)	0 (0.0%)	2 (1.6%)
JAK2 allelic burden in % (IQR)	26.5 (11.3 - 42.1)	48.8 (17.1 - 76.9)	44.0 (22.2 - 55.6)	76.4 (19.3 - 90.7)	34.5 (12.5 - 60.8)
Presence of blasts on presentation	1 (1.6%)	0 (0.0%)	4 (36.4%)	1 (14.3%)	6 (4.7%)
Signs & Symptoms					
MPN score (min-max range)	0 (0 - 2)	0 (0 - 3)	0 (0 - 3)	0 (0 - 2)	0 (0 - 3)
Splenomegaly	5 (8.1%)	11 (22.9%)	8 (72.7%)	3 (42.9%)	27 (21.1%)
Treatment & Complications					
On hydroxyurea (HU) regime	40 (64.5%)	35 (72.9%)	1 (9.1%)	2 (28.6%)	78 (60.9%)
Thrombotic event(s) in the past	26 (41.9%)	28 (58.3%)	3 (27.3%)	3 (42.9%)	60 (46.9%)

**Table 3:** Haematological Associations for the Total Combined Cohort, ET and PV Patients

Haematological associations for the total combined cohort					
Laboratory Marker	MWU	Mean rank (≤50%)	Mean rank (>50%)	N	p
WBC	1016.5	56.8	81.5	127	0.001
Hb	1287	59.8	74.2	127	0.045
Platelets	1164	69.6	50.5	127	0.008
LDH	611.5	41.7	62.5	97	0.001
Haematological associations for ET patients					
Laboratory Marker	MWU	Mean rank (≤50%)	Mean rank (>50%)	N	p
WBC	141	30.6	38.9	62	0.252
Hb	134	32.6	23.1	62	0.193
Platelets	184	31.3	32.7	62	0.85
LDH	113	21.6	23.8	43	0.669
Haematological associations for PV patients					
Laboratory Marker	MWU	Mean rank (≤50%)	Mean rank (>50%)	N	p
WBC	137.5	18.2	30	47	0.003
Hb	166.5	19.4	28.8	47	0.02
Platelets	223	26.2	21.7	47	0.259
LDH	56	12	23.7	36	0.001

## Discussion

The differences in the ET and PV cohorts were largely expected based on disease profile. Specifically, PV patients tended to have a greater incidence of splenomegaly, higher Hb counts and lower platelet counts compared to the ET patients. In a Thailand-based study done by Singdong et al., (2016), higher mutant allelic loads were seen in PV compared to ET. The same trend was observed in this study, with ET patients having the lowest percentage allelic burden, followed by PMF, PV, and MPN-NOS in ascending order.

The mechanism behind how different allelic burdens of the same mutation can give rise to the different MPN phenotypes may lie in miRNA deregulations. Recent studies suggest that miRNAs deregulations may be the downstream targets of abnormal JAK2 signalling (Zhan et al., 2013). At the progenitor cell level, the developmental fate of the megakaryocyte-erythroid progenitor (MEP) cell, is regulated in part by miRNAs (Lu et al., 2008). The variation in allelic burden is associated with miRNA deregulation and thus potentially determines phenotype; a low allelic burden, for example, commits MEPs toward megakaryocyte differentiation at the expense of erythrocytes, and vice versa.

The systematic review by Vannuchi et al., (2011) showed that higher allelic burdens in MPN were associated with splenomegaly, consistent with our findings. The review also reported that higher allelic burdens are associated with more symptoms, especially in the context of PV, thrombotic events, especially in the context of ET, and initiation of cytoreductive treatment in the context of both. Both chi-squared test of independence and Spearman’s correlation for the aforementioned areas in this study yielded insignificant results. This could be due to the fact that the MPN10 score was based on self-reported symptoms, and the cohort may not be large enough to demonstrate a conclusive trend in these areas. Moreover, MPN symptoms may not be as common in the Asian cohort. The reported symptoms scored a median of zero throughout all MPN subtypes. It is known that there are ethnicity differences in MPN with regards risk of thrombotic and haemorrhagic events, as well as progression to myelofibrosis (Khan et al., 2016). However, there has been no published study in terms of symptomology complex.

In terms of haematological parameters, in the combined cohort analysis, it was shown that higher allelic burdens are associated with higher cell counts and LDH levels. In the PV subgroup, higher allelic burden was significantly related to higher WBC and Hb counts, and was inversely associated with platelet counts, although not statistically significant (p = 0.259). This inverse relationship between allelic burden and platelet count is unique to PV patients. Although this phenomenon in our cohort did not reach statistical significance, it has been reported by others in the literature (Passamonti et al., 2010). A previous study in murine cells suggests that low levels of JAK2 kinase activity favour thrombocytosis whereas high levels favour erythrocytosis, possibly accounting for this inverse relationship (Lacout et al., 2006).

Higher allelic burdens did not always correlate with predictable haematological parameters in our study. In the ET subgroup, allelic burden was not significantly associated with any of the haematological parameters of LDH, WBC count, Hb and platelet levels. In several studies, including three retrospective studies done by Larsen et al. (2007), Kittur et al., (2007) and Antonioli et al., (2008), the allelic burden seemed to correlate positively with WBC and Hb counts and negatively with platelet counts in patients with ET. In other studies, however, such as the retrospective studies by Singdong et al., (2016) and Ha et al., (2012), and in our cohort, these trends were not significant. One interesting observation from the studies above is the ethnic difference between the populations studied. The Western population appeared to correlate with haematological parameters and allelic burden whereas the studies in Eastern patients did not. It is unclear if there is an ethnic difference in JAK2 positive ET patients but this bears further investigation. 

With regards to thrombotic complications, our study is consistent with the literature in that arterial events (cerebrovascular accident, acute coronary syndrome, peripheral arterial thrombosis) are more common than venous thrombosis (splanchnic thrombosis, deep vein and pulmonary embolism) in the context of MPN (McMahon and Stein, 2013). Relative differences, although not statistically significant in this study, follow that of the literature; highest thromboses rates were noted in the PV group, followed by ET and PMF in that order.

As for co-existing mutations, it is known from the literature that double-mutation cases are uncommon, with a reported incidence of 3-5% (Usseglio et al., 2017; Nessenzveig et al., 2016; Haunstrup et al., 2018), similar to our cohort’s statistic of 2.34%. However, double-mutations are often limited to cases with low JAK2 allelic burden, which was not seen in our study in the two patients with CALR positive mutations. 

Survival analysis showed no significant difference between the groups with low and high JAK2 allelic burden respectively. We hypothesise this could be due to two reasons. Firstly, treatment choice and intensity is tailored to disease severity and this likely alters the prognosis of the disease, especially those with high allelic burden, in the short term. Secondly, the follow-up period is a relatively short duration of three years. This implies a significant number of right-censored cases where the mortality event has not been reached. Future studies could compare the long-term survival distributions between the two groups.

In conclusion, our study shows that Asian MPN patients’ results are in concordance with previous research demonstrating the association between allelic burden and clinico-haematological manifestations of MPN. The observation that Asian ET patients behave differently to Western patients warrants further study.

Other future directions include prospective studies to establish a causal relation of JAK2 mutations with MPN phenomena, examining the role of co-existing mutations such as TET2 and investigating the allelic burden response to response to treatment. 
